# Reporting methodological issues of the mendelian randomization studies in health and medical research: a systematic review

**DOI:** 10.1186/s12874-022-01504-0

**Published:** 2022-01-16

**Authors:** Shabab Noor Islam, Tanvir Ahammed, Aniqua Anjum, Olayan Albalawi, Md. Jamal Uddin

**Affiliations:** 1 Department of Statistics, Shahjalal University of Science and Technology, 3114 Sylhet, Bangladesh; 2 Department of Statistics, Faculty of Science, University of Tabuk, Tabuk, Saudi Arabia

**Keywords:** Instrumental variable analysis (IV), Mendelian randomization, Genetic risk scores, Systematic review

## Abstract

**Background:**

Mendelian randomization (MR) studies using Genetic risk scores (GRS) as an instrumental variable (IV) have increasingly been used to control for unmeasured confounding in observational healthcare databases. However, proper reporting of methodological issues is sparse in these studies. We aimed to review published papers related to MR studies and identify reporting problems.

**Methods:**

We conducted a systematic review using the clinical articles published between 2009 and 2019. We searched PubMed, Scopus, and Embase databases. We retrieved information from every MR study, including the tests performed to evaluate assumptions and the modelling approach used for estimation. Using our inclusion/exclusion criteria, finally, we identified 97 studies to conduct the review according to the PRISMA statement.

**Results:**

Only 66 (68%) of the studies empirically verified the first assumption (Relevance assumption), and 40 (41.2%) studies reported the appropriate tests (e.g., R2, F-test) to investigate the association. A total of 35.1% clearly stated and discussed theoretical justifications for the second and third assumptions. 30.9% of the studies used a two-stage least square, and 11.3% used the Wald estimator method for estimating IV. Also, 44.3% of the studies conducted a sensitivity analysis to illuminate the robustness of estimates for violations of the untestable assumptions.

**Conclusions:**

We found that incompleteness of the justification of the assumptions for the instrumental variable in MR studies was a common problem in our selected studies. This may misdirect the findings of the studies.

**Supplementary Information:**

The online version contains supplementary material available at 10.1186/s12874-022-01504-0.

## Background

Understanding the causal associations between outcome and exposures is crucial in the health and medical sciences for various reasons, including preventive measures, advanced detection and intervention, and better treatment and support. Observational studies are the most effective approach to investigate the causal relationships between exposures and outcomes since randomized controlled trials (RCTs) studies are often ethically or practically unfeasible [1]. However, these relationships may be confounded by associated components if treatments are not assigned at random [2–4]. Therefore, analytical approaches which can minimize bias and evaluate the causal effects in the presence of confounding that are not measured in observational studies can offer more convincing confirmation of causal inference. An instrumental variable (IV) analysis is a technique for obtaining consistent causal estimates in the presence of unobserved confounding [4, 5]. Usually, an instrumental variable, also known as an “instrument,” is a variable that has a relationship with exposure of interest, i.e., exogenous variable, but does not have an association with the outcome, i.e., endogenous variable, except in the context that it influences the exposure, which in turn affects the endogenous variable [6, 7]. Though an instrumental variable can be any trait that meets these criteria, the genetic variants are strong candidates for instrumental variables [2, 8]. This is because genetic variations are generally inherited independently, and more importantly, they are unlikely to be affected by confounding variables as they are predetermined [2, 3]. For the last ten years, this approach of treating genetic variants as instrumental variables in observational data to explore the consequences of changeable risk factors for diseases has been termed ‘Mendelian randomization’ (MR) [3, 9, 10]. Genetic risk scores (GRS), also called polygenic risk scores (PRS), genotype scores, gene scores, or allele scores, are a more straightforward means of summing up an enormous amount of genetic variants correlated with a potential cause.

The GRS, usually based on genome-wide single-nucleotide polymorphism (SNP) data, is constructed using a set of SNPs discovered in a discovery genome-wide association studies (GWAS) (usually from a different training sample) [11–15]. An unweighted score is calculated using the total number of risk factor-increasing alleles in a person’s genotype. On the other hand, in a weighted score, a weight is assigned to each allele depending on the impact of the related genetic variation on the risk factor. These weights might be calculated internally from the examined data or externally from prior information or a separate data source. In this approach, multidimensional genetic variations associated with a risk factor can be reduced to a single variable and used in a Mendelian randomization study under the assumption that the GRS is an instrumental variable [16].

MR studies must satisfy the assumptions of the instrumental variable since genetic variants are used as an instrumental variable in these studies. These assumptions for MR studies are [1, 8, 17, 18]:

(i) Relevance assumption: There is an association between the genetic variants and the exposure. Even though the assumption simply needs the existence of an association, weak associations provide little statistical power for testing hypotheses and amplify the bias resulting from violations of the instrumental variable assumptions. F-statistics, R square, odds ratio, or the risk difference are usually used to assess the association.
(ii) Exclusion restriction assumption: The influence of genetic variants on the exposure of interest is the only way through which they affect the outcome. More simply, genetic variants are not directly associated with the outcome, but they do influence the exposure, and exposure affects the outcome. This assumption can be assessed by detecting horizontal pleiotropy.
(iii) Independence assumption: This assumption is also known as the exchangeability assumption. According to this assumption, there is no confounding for the effect of genetic variants on the outcome. It may also be stated as the instruments do not share any causes with the outcome. The third assumption can be assessed by checking for correlations between the genetic instrument and common confounders, bias component plots, covariate balance tests, adjustment for principal components of population stratification, and evidence from large GWAS on the association of the genetic variants used as instruments with other baseline factors [8].

An overidentification test, i.e., the Sargan or the Hansen test [19, 20], can be performed to determine if the parameters calculated by each IV individually are similar when using several instruments [21]. Failure of the test reveals variability in the effect estimates from each IV, implying that one or more genetic variants may violate IV assumptions. However, it is not possible with a single instrument [22].

The first three assumptions simply define the causal effect’s bounds independently derived by Robins and Manski (later Balke and Pearl derived smaller bounds) [23–27]. Thus, a fourth identifying assumption is often not mentioned and is required to obtain a point estimate [1, 4, 27, 28]. The assumption is based on effect homogeneity, which states that exposure’s effect on outcome should be consistent across the subjects. It is, nevertheless, infeasible. As a result, an alternative assumption that does not need effect homogeneity has been established. This assumption is known as the monotonicity assumption or no defiers. For example, in a clinical setting, we can say that there are no defiers if no patients would be recommended treatment A when consulted by a doctor who generally recommends treatment B and would be suggested B by a doctor who normally suggests treatment A [27, 29]. In other words, according to this assumption, the proposed IV must only affect exposure in one direction, i.e., there should not be cases where the exposure level is increased by increasing the proposed IV and cases where the exposure level is decreased by increasing the proposed IV [18, 30]. In addition, the causal parameter of interest depends on the choice of this assumption[18]. For example, the homogeneity assumption (4 h) for estimating the Average Treatment Effect (ATE) and the monotonicity assumption (4 m) for estimating the Local Average Treatment Effect (LATE) is needed to be theoretically justifiable [5, 18, 31].

The goal of MR studies can be achieved only when the assumptions are met, and the authors provide adequate evidence for reviewers and readers to evaluate [6, 27] and to assess the efficacy of analysis in Mendelian randomization studies, the assumptions must be presented. Studies, however, have shown that there is insufficient reporting of the credibility of MR assumptions as well as the statistical methods applied in MR studies [2]. Inadequate reporting of methodologies, validation of the assumptions, and sensitivity analyses can affect the result and the utilization of the study data. These problems may also lead the authors of MR studies to biased or false conclusions [32]. Therefore, in this study, we focus on evaluating if the researchers have explained the assumptions of the MR studies. Additionally, we assessed if the applied statistical methods have adequately been defined, along with the derivation of the confidence interval for those studies.

## Methods

We adopted the Preferred Reporting Items for Systematic Review and Meta-Analysis Protocols (PRISMA-P) 2009 [33].

### Search strategy

To evaluate the effectiveness of the research regarding the instrumental variables, we performed a systematic review using the studies published from 2009 to 2019 in PubMed, Scopus, and Embase. We searched articles for MR studies where the GRS is used as a covariate. The search terms were: “Mendelian randomization” AND “allele scores”, OR “genetic risk scores” OR “polygenic risk score” OR “gene scores”, OR “genotype scores”, OR “GRS”, OR “PRS”. We also checked the reference lists of the included articles and reached out to experts. To eliminate duplicates and to handle the records, Mendeley version 1.19.8 software was used.

### Inclusion and exclusion criteria

We selected an article that reported Mendelian Randomization and polygenic risk score, or genetic risk score based on either individual level data or two-sample summary-data; used minimum 500 samples, and published in English in any country or region in the world. Reviews articles, short communications, editorials, case reports, letters to the editor were not considered in this study. Moreover, two papers were excluded as they used SNP as GRS.

### Data screening and extraction

Following the duplicate articles’ removal, we screened the titles and abstracts and then assessed the remaining full-text articles for inclusion. Discussions with co-authors were used to settle the differences of opinion. Data from all eligible studies were extracted using a standardized form. Information about instrumental variables, including tests performed to assess the assumptions, were extracted for each included paper. A total of 97 research articles were included.

### Software

For the data analyses, we used SPSS (v25) and Excel 2019.

## Results

**Fig. 1:**
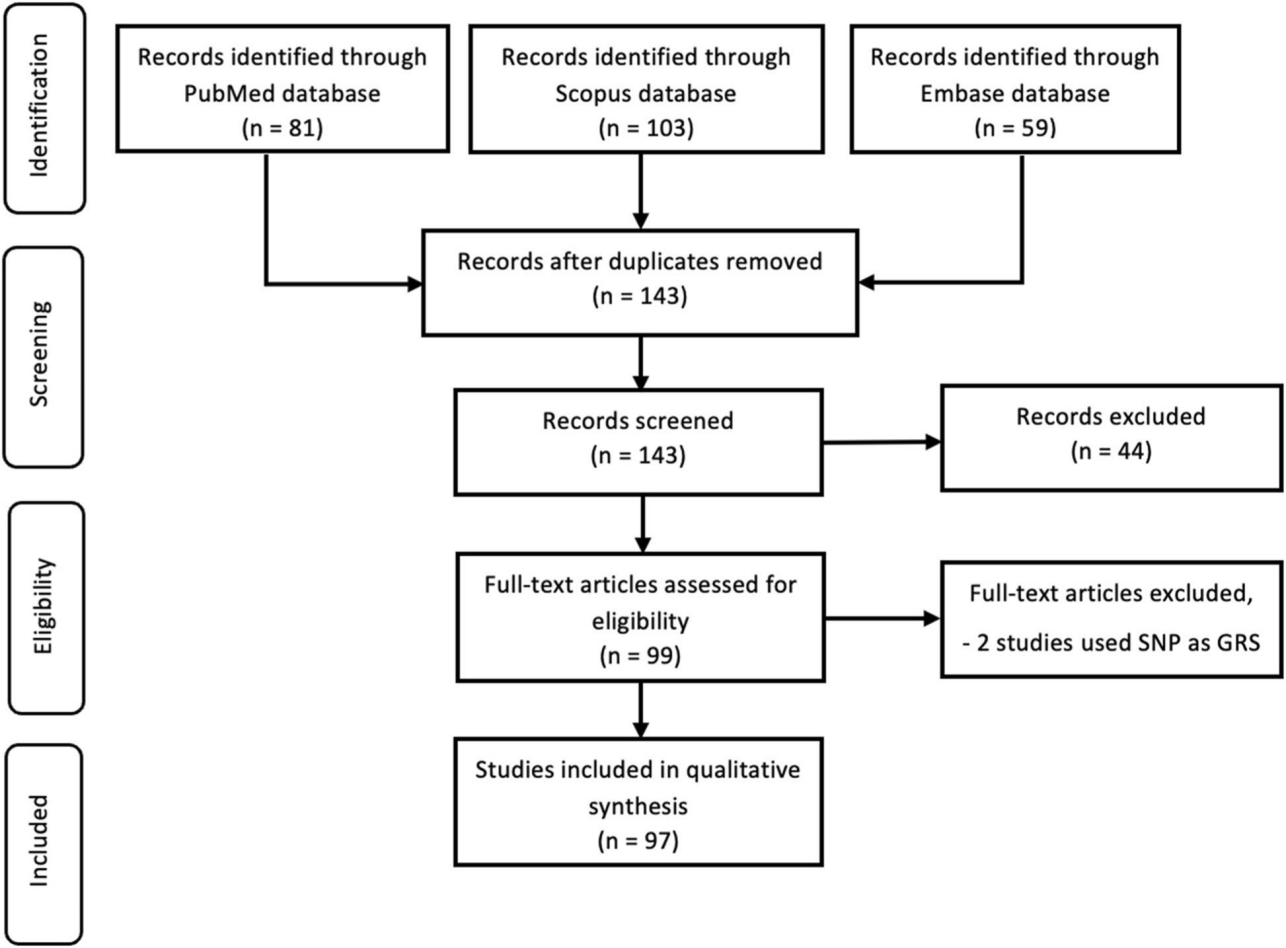
Flow diagram for the studies included in the systematic review

At first, we identified 143 unique studies. We reviewed all these identified studies and excluded 44 meta-analysis studies because of our exclusion criteria and selected 99 articles for further investigation. Out of these 99 articles, we found that two studies had used a single SNP as GRS. Finally, after excluding those two studies, we included 97 studies for the review (Fig. 1) and 16.49% of our reviewed articles used two sample MR approach.

Table 1 presents how the studies included in the review described the steps for reporting MR studies. The systematic review identified 32.0% included studies overlooked the first assumption. Furthermore, 40.2% of the studies had not provided any information regarding both the second and third assumptions. Moreover, only one study reported more than one type of falsification tests for the second and third assumption and 47.4% study investigate directional pleiotropy. Only 8.2% of studies clearly stated the treatment effect to be estimated, though 81.4% of studies did not report the estimated bounds for the casual effect under 1st, 2nd, and 3rd assumption. There was no theoretical explanation for the fourth assumption in approximately 89.7% of studies. A total of 44.3% of the studies conducted a sensitivity analysis, and 25.8% of studies discussed linkage disequilibrium.

**Table 1:** Percentage Reporting According to Suggested Guidelines in a Review of IV Publications Assessing Effects of Medical Interventions (*n* = 97)

Guideline	Count	Percentage
Empirically verified 1st assumption		
Yes	66	68.0
No	31	32.0
Strength of the 1st assumption		
Verified in data using F-statistic	28	28.9
Verified in data using F-statistic and R^2^	11	11.3
Verified in data using odds ratio	1	1.0
Not reported	57	58.8
Provided theoretical justifications for 2nd and 3rd assumption		
Clearly Stated & Discussed	34	35.1
Lacked Clear Discussion	24	24.7
No Acknowledgment	39	40.2
Clearly reported falsification tests for 2nd and 3rd assumption		
Reported two or more types	1	1.0
Reported exactly one type	7	7.2
Did not report any tests	89	91.8
Detection of pleiotropy		
Yes	46	47.4
No	51	52.6
Clearly stated the effect to be estimates		
The effect in the population (Average treatment effects, ATE)	1	1.0
Effect in the compliers (Local average treatment effects, LATE)	6	6.2
Both stated (ATE & LATE)	1	1.0
Not stated	89	91.8
Estimated causal effect bounds, under the 1st, 2nd, and 3rd assumption		
Yes	18	18.6
No	79	81.4
Discussed theoretical justification for the pertinent fourth assumption		
Stated and discussed homogeneity assumption (4 h)	1	1.0
Stated and discussed monotonicity assumption (4 m)	4	4.1
Stated and discussed both (4 h) and (4 m)	0	0.0
Stated but not discussed (4 h)	3	3.1
Stated but not discussed (4 m)	2	2.1
No acknowledgment of the 4th assumption	87	89.7
Modeling approach for the estimation was clearly described		
The modeling approach clearly described	74	76.3
Lack of adequate description of the modeling approach	23	23.7
Conduct Sensitivity Analysis		
Yes	43	44.3
No	54	55.7
Discussed Linkage Disequilibrium		
Yes	25	25.8
No	72	74.2

A total of 30.9% of the studies used a two-stage least square method to estimate IV, whereas 11.3% of the studies used a Wald estimator for evaluation of the parameter. Moreover, 24.7% studies used inverse variance weighted method for estimating the parameters from IV models (Table 2).

**Table 2:** Frequency of the modeling approach

Model Name	Count	Percentage
Two-stage least square (2SLS)	30	30.9
Inverse Variance Weighted Method (IVW)	24	24.7
Wald Estimator	11	11.3
Two-stage residual inclusion (2SRI)	2	2.1
Bivariate probit method (BPM)	2	2.1
2SLS and IVW	2	2.1
IVW and Wald Estimator	2	2.1
Limited information maximum likelihood (LIML)	1	1.0

## Discussion

In this study, we evaluated the reporting problems of GRS as an instrumental variable in MR studies. Overall, consistent with previous studies [1, 2], we found that many studies did not report an adequate amount of information. Which lead to the problem of determining if the authors’ inferences were supported by their evidence. Though only the first assumption or the relevance assumption can be empirically verified, about two-third (68.0%) of the included studies reported checking this assumption [30]. However, less than half of the studies reported the appropriate tests for empirical verification of the 1st assumption. Moreover, almost all these studies reported an F-statistic or both the F-statistic and R2 for empirical verification of the 1st assumption.

According to the first assumption, a weak association between the GRS and exposure can intensify biases caused by slight violations of the second or third assumption, resulting in biased estimates [34, 35] and provide little statistical power to test hypotheses [8]. On the other hand, an extremely strong association would be far more likely to violate the second or third assumption. Moreover, the GRS is suspected to be linked with about the same group of confounding variables (possibly unmeasured) as the exposure if the correlation is perfect [28]. Furthermore, while the first stage F-statistics is a well-established statistic for measuring instrument strength [36], providing both the F-statistic value and the association between exposure and IV using Pearson’s correlation, Odds Ratio, or point bi-serial correlation is suggested [37].

In our review, it was found that more than one-third of the studies did not even mention the theoretical justification for the second and third assumptions. As opposed to the first assumption, second and third assumptions are not experimentally verifiable. Hence, an analyst uses subject-matter expertise to develop a case for why the offered instrument is considerately supposed to follow both assumptions. Even if the second and third assumptions cannot be proven to be true, it is frequently possible to falsify them [5, 38]. Regarding falsification tests for the 2nd and 3rd assumption, a negligible proportion (only 1.0%) reported two or more tests, and seven studies (7.2%) reported exactly one test. However, almost half of these studies investigate directional pleiotropy which can be used to assess the third assumption [1].

Under the first, second, and third assumptions, about one-fifth of studies estimated causal effect bounds. The importance of these bounds is that they indicate how much information is needed to fill in the as well as how much information is required to be given by a fourth assumption to express the inaccuracy regarding the causal effect when the data and all three assumptions are combined [5, 6, 27].

It is needed to determine the causal effect of interest in estimating both bounds and effects. The average treatment effect (ATE) in the population and the local average treatment effect (LATE) in the subpopulation are the two main options for determining the causal effect of interest. In general, the ATE and LATE can vary, so the analyst should define the purposes for picking one over another. Just one study out of ten in our analysis was explicit, and the majority of the studies did not mention anything about this topic.

Our review found that a majority of the studies (89.7%) did not acknowledge any fourth assumption, while about 4% stated and discussed homogeneity or a monotonic effect. As the choice of the causal effect of interest, i.e., ATE, LATE, depends on the theoretical justification of the (4 h) or (4 m) assumption, respectively, calculating effect estimates may be appropriate if the first, second, and third assumptions, as well as either (4 m) or (4 h), are entirely justified [5, 18, 31]. Models that approximate these effects inside levels of calculated covariates can also be used, but the necessary assumptions must hold conditional on these covariates. Most of the studies stated the modeling approach for estimating the parameter from IV models. Sensitivity analyses are used to illuminate the robustness of estimates for violations of the untestable assumptions. Furthermore, pleiotropy-tolerant MR techniques are sometimes referred to as sensitivity analyses. However, its implementation seldom includes implicit falsification tests, such as the MR-Egger intercept, which may be used to test for violations of the exclusion restriction assumption. It was found that more than half of the studies did not conduct sensitivity analysis.

Another common technical problem for MR analyses is linkage disequilibrium which is relevant to the MR assumptions. However, twenty-five, i.e., 25.8% of the articles neither discussed this issue nor the possible impacts on the results.

While checking the standards of the study based on fulfilling the main three assumptions, we found that more than two-third (71.1%) of the included studies are not standard. About 30% of the studies fell into the standard category, i.e., these studies mentioned the assumptions, provided the empirical and theoretical justifications, investigated horizontal pleiotropy and reported falsification tests for the assumptions.

Lor et al. recently defined and assessed the reporting of MR analyses. They did, however, solely look at oncological studies. Over half of the literature (51.9%) they reviewed did not mention the first three MR assumptions, and 14% of studies had inadequately stated procedures for IV analysis [1]. Boef et al. reviewed existing MR literature concentrating on the methodological procedures utilized in MR research, as well as discussion of the assumptions and reporting of the statistical methods used. However, they included studies up to December 2013. According to their findings, less than half of the papers (44%) addressed the plausibility of all three MR assumptions [2].

The MR analyses field has evolved substantially in recent years as many different tools and techniques are available for carrying out MR studies comparing to the past. Therefore, updated knowledge is necessary to check if newer MR articles are more likely to follow the MR analysis criteria. As a result, we included articles up to 2020 and split them into two categories based on whether or not the publication was published before 2017. However, we have failed to identify any significant reporting quality difference (P-value = 0.746) in the current MR studies, i.e., studies published in 2017 and later indicating that reporting quality of MR studies are still not up to the mark.

As a significant amount of the included studies did not report sufficient information, we suggest a checklist of information and specification tests for the investigators of MR studies:

- State explicitly the four MR assumptions along with any additional or sensitivity analysis assumptions.
- Describe any methods applied to evaluate or explain the assumptions’ validity in the study, as well as the possible effect of assumption violation and the evaluation and reduction of potential biases due to assumption violation.
- Discuss the MR estimator, such as two-stage least squares, two-stage residual inclusion, Wald ratio, bivariate probit method, or limited information maximum likelihood and related statistics.
- State the estimated causal effect between outcome and exposure, as well as report the MR analysis results with confidence intervals.
- Explain any sensitivity analyses or other analyses that were performed.
- Specify the genetic instrument’s strength and address the limitations of the study, considering sources of potential bias (i.e., linkage disequilibrium).
- Follow STROBE-MR: Guidelines for strengthening the reporting of Mendelian randomization studies [39].

## Conclusions

We found that incompleteness of the justification for the assumptions of the GRS as an instrumental variable was a common problem in our selected studies. This may misdirect the quality of the study in the wrong way. So, we point out that the fundamental issue in MR studies is not the decision of technique but instead the selection of appropriate GRS as IV and the evaluation of the IV assumptions. Therefore, we recommend routinely evaluate and justify the assumptions.

## Supplementary Information

**Additional file 1.**

## Data Availability

The complete list of extracted data from all included studies are provided in the paper (Additional file 1). No additional supporting data is available.

## Abbreviations

MR: Mendelian Randomization
IV: Instrumental Variable
GRS: Genetic Risk Scores
PRS: Polygenic Risk Scores
ATE: Average Treatment Effect
LATE: Local Average Treatment Effect
