# Supplementary material for: Reporting methodological issues of the mendelian randomization studies in health and medical research: a systematic review
Source: BMC Med Res Methodol. 2022 Jan 16;22:21. doi: 10.1186/s12874-022-01504-0 (PMC8761268; doi:10.1186/s12874-022-01504-0)
Supplement: Supplementary file 1 — Additional file 1. [file 12874_2022_1504_MOESM1_ESM.docx]

**Appendix**

**Additional file 1**. Characteristics of the included studies in the systematic review.

| Study | Empirically verified 1st assumption | Strength of the 1st assumption | Provided theoretical justifications for 2nd and 3rd assumptions | Reported falsification tests for 2nd and 3rd assumptions | Clearly stated the effect to be estimates | Estimated bounds for the causal effect, under 1st, 2nd, and 3rd assumptions | Discussed theoretical justification for the pertinent fourth assumption | modeling approach used for estimation | Exposure effect | Covariate adjustment | Sensitivity Analysis | Linkage Disequilibrium |
| --- | --- | --- | --- | --- | --- | --- | --- | --- | --- | --- | --- | --- |
| Allard et al., 2015 | No |  | No Acknowledgment | Not clearly reported | Not stated | No | No Acknowledgment | 2SLS | Regression coefficient | Unadjusted | No | Not Stated |
| Allin et al, 2016 | Yes | F | No Acknowledgment | Not clearly reported | Not stated | No | No Acknowledgment | 2SLS | Regression coefficient | Adjusted | Yes | Not Stated |
| Amini et al., 2018 | Yes | F | No Acknowledgment | Not clearly reported | Not stated | No | No Acknowledgment | 2SLS | OR | Adjusted | Yes | Not Stated |
| Archangelidi et al., 2017 | No |  | No Acknowledgment | Not clearly reported | Not stated | No | No Acknowledgment |  | Regression coefficient | Adjusted | Yes | Not Stated |
| Benn et al., 2012 | Yes | F | No Acknowledgment | Not clearly reported | Not stated | No | No Acknowledgment | 2SLS | OR | Adjusted | No | Not Stated |
| Bockerman et al., 2018 | Yes | F | Clearly Stated & Discussed | Not clearly reported | LATE | No | Stated and Discussed 4h |  | Regression coefficient | Unadjusted | No | Not Stated |
| Bonilla et al., 2016 | Yes |  | Lacked Clear Discussion | Not clearly reported | Not stated | No | No Acknowledgment |  | OR | Adjusted | No | Stated |
| Broek et al., 2018 | No |  | Lacked Clear Discussion | Not clearly reported | Not stated | No | No Acknowledgment | IVW | OR | Adjusted | Yes | Not Stated |
| Buddu-Aggrey et al., 2019 | No |  | No Acknowledgment | Not clearly reported | Not stated | Yes | No Acknowledgment | 2SLS | OR | Adjusted | No | Not Stated |
| Burgess et al., 2015 | Yes | F | Lacked Clear Discussion | Not clearly reported | LATE | No | Stated but not Discussed 4m | 2SLS, IVW | Regression coefficient | Unadjusted | No | Not Stated |
| Censin et al., 2017 | Yes |  | Lacked Clear Discussion | Not clearly reported | Not stated | No | No Acknowledgment | IVW | OR | Unadjusted | Yes | Not Stated |
| Censin et al., 2019 | Yes | F | Lacked Clear Discussion | Not clearly reported | Not stated | Yes | No Acknowledgment | Wald Estimator, IVW | OR | Adjusted | Yes | Not Stated |
| Chatterjee et al., 2016 | No |  | No Acknowledgment | Not clearly reported | Not stated | No | No Acknowledgment | IVW | HR | Adjusted | No | Not Stated |
| Chen et al., 2018 | Yes | F, R^2^ | Clearly Stated & Discussed | Not clearly reported | Not stated | Yes | No Acknowledgment | Wald Estimator | Regression coefficient | Unadjusted | Yes | Not Stated |
| Chen et al., 2019 | Yes | F, R^2^ | Clearly Stated & Discussed | Not clearly reported | Not stated | Yes | No Acknowledgment |  | Regression coefficient | Adjusted | No | Not Stated |
| Chen et al., 2018 | No |  | Lacked Clear Discussion | Not clearly reported | Not stated | No | No Acknowledgment |  | OR | Unadjusted | No | Stated |
| Chen et al., 2019 | Yes | F | Lacked Clear Discussion | Not clearly reported | Not stated | Yes | No Acknowledgment | Wald Estimator | OR | Adjusted | Yes | Not Stated |
| Davies et al., 2015 | No |  | No Acknowledgment | Not clearly reported | Not stated | No | No Acknowledgment |  | OR | Unadjusted | Yes | Stated |
| DiPrete et al., 2018 | Yes |  | Clearly Stated & Discussed | Not clearly reported | Not stated | No | Stated and Discussed 4m | 2SLS | Regression coefficient | Adjusted | No | Not Stated |
| Esmeijer et al., 2019 | No |  | No Acknowledgment | Not clearly reported | LATE | No | Stated and Discussed 4m | IVW | OR | Adjusted | No | Not Stated |
| Fall et al., 2013 | Yes |  | Lacked Clear Discussion | Reported 1 type | Not stated | No | Stated and Discussed 4m | 2SLS | Regression Coefficient | Adjusted | Yes | Stated |
| Gao et al., 2016 | No |  | Clearly Stated & Discussed | Not clearly reported | Not stated | No | No Acknowledgment | Wald Estimator | OR | Adjusted | No | Not Stated |
| Gianfrancesco et al., 2017 | No |  | No Acknowledgment | Not clearly reported | Not stated | No | No Acknowledgment |  | OR | Unadjusted | No | Not Stated |
| Gianfrancesco et al., 2017 | No |  | No Acknowledgment | Not clearly reported | Not stated | No | No Acknowledgment |  | OR | Adjusted | No | Not Stated |
| Gkatzionis et al., 2018 | No |  | No Acknowledgment | Not clearly reported | Both | No | No Acknowledgment | Wald Estimator | OR | Adjusted | No | Not Stated |
| Guo et al., 2019 | Yes |  | Clearly Stated & Discussed | Not clearly reported | Not stated | No | No Acknowledgment | IVW | OR | Unadjusted | No | Not Stated |
| Hamad et al., 2016 | No |  | No Acknowledgment | Not clearly reported | LATE | No | Stated but not Discussed 4m | IVW | OR | Adjusted | No | Not Stated |
| He et al., 2018 | Yes |  | No Acknowledgment | Not clearly reported | Not stated | No | No Acknowledgment | 2SLS | Regression coefficient | Adjusted | Yes | Not Stated |
| Holmes et al., 2014 | Yes | F | Clearly Stated & Discussed | Reported 1 type | Not stated | No | No Acknowledgment | 2SLS | Regression Coefficient, OR | Adjusted | Yes | Not Stated |
| Huang et al., 2016 | No |  | Lacked Clear Discussion | Not clearly reported | Not stated | No | No Acknowledgment | Wald Estimator | OR | Adjusted | Yes | Stated |
| Huang et al., 2019 | Yes | F | Clearly Stated & Discussed | Not clearly reported | Not stated | Yes | No Acknowledgment | IVW | OR | Adjusted | Yes | Stated |
| Hughes et al., 2014 | Yes | F | Clearly Stated & Discussed | Not clearly reported | Not stated | No | No Acknowledgment | 2SLS | Regression coefficient | Unadjusted | No | Not Stated |
| Hung et al., 2014 | Yes | F | No Acknowledgment | Not clearly reported | Not stated | No | No Acknowledgment | BPM | Regression coefficient | Adjusted | No | Not Stated |
| Islam et al., 2012 | No |  | No Acknowledgment | Not clearly reported | Not stated | Yes | No Acknowledgment | 2SRI | RR | Adjusted | Yes | Not Stated |
| Jee et al., 2019 | Yes | F | Lacked Clear Discussion | Not clearly reported | Not stated | No | No Acknowledgment | 2SLS | OR | Adjusted | Yes | Stated |
| Jia et al., 2019 | Yes |  | Clearly Stated & Discussed | Not clearly reported | Not stated | Yes | No Acknowledgment | IVW | Regression coefficient | Adjusted | Yes | Stated |
| Jokela et al., 2012 | Yes | F | Clearly Stated & Discussed | Not clearly reported | Not stated | No | No Acknowledgment |  | OR | Adjusted | No | Not Stated |
| Kim et al., 2018 | No |  | No Acknowledgment | Not clearly reported | Not stated | No | No Acknowledgment | 2SLS | OR | Unadjusted | No | Not Stated |
| Kleber et al., 2015 | No |  | No Acknowledgment | Not clearly reported | Not stated | No | No Acknowledgment | 2SLS | HR | Unadjusted | No | Stated |
| Lee et al., 2018 | Yes | F | Clearly Stated & Discussed | Not clearly reported | Not stated | No | No Acknowledgment | Wald Estimator | OR | Adjusted | No | Not Stated |
| Li et al., 2016 | No |  | No Acknowledgment | Not clearly reported | Not stated | No | No Acknowledgment |  | Regression coefficient | Unadjusted | No | Not Stated |
| Liu et al., 2015 | Yes | F, R^2^ | Lacked Clear Discussion | Not clearly reported | Not stated | No | No Acknowledgment | 2SLS | Regression Coefficient | Unadjusted | No | Not Stated |
| Machiela et al., 2014 | No |  | Lacked Clear Discussion | Not clearly reported | Not stated | No | No Acknowledgment |  | OR | Unadjusted | No | Not Stated |
| Magnus et al., 2018 | Yes | F, R^2^ | Clearly Stated & Discussed | Not clearly reported | Not stated | Yes | No Acknowledgment | IVW | RR | Unadjusted | No | Stated |
| Magnus et al.,2018 | Yes | F | Lacked Clear Discussion | Reported 1 type | Not stated | Yes | No Acknowledgment | IVW | RR | Adjusted | Yes | Not Stated |
| Mao et al., 2017 | Yes |  | Clearly Stated & Discussed | Not clearly reported | Not stated | No | No Acknowledgment | Wald Estimator, IVW | OR | Unadjusted | Yes | Stated |
| Mehendran et al., 2017 | No |  | No Acknowledgment | Not clearly reported | Not stated | No | No Acknowledgment | 2SLS | Regression coefficient | Adjusted | No | Not Stated |
| Merino et al., 2017 | No |  | No Acknowledgment | Not clearly reported | Not stated | No | No Acknowledgment | IVW | OR | Unadjusted | No | Stated |
| Millard et al., 2015 | Yes | F | Clearly Stated & Discussed | Not clearly reported | Not stated | Yes | Stated but not Discussed 4h | 2SLS | Regression coefficient | Adjusted | Yes | Stated |
| Millard et al., 2019 | Yes | F | Lacked Clear Discussion | Not clearly reported | Not stated | No | No Acknowledgment | BPM | OR | Adjusted | Yes | Not Stated |
| Nguyen et al., 2016 | Yes |  | Clearly Stated & Discussed | Not clearly reported | Not stated | No | No Acknowledgment |  | Regression coefficient | Adjusted | Yes | Not Stated |
| Nishiyama et al., 2019 | Yes | F | No Acknowledgment | Not clearly reported | Not stated | No | No Acknowledgment | 2SLS, IVW | Regression coefficient | Adjusted | Yes | Not Stated |
| Oikonen et al., 2012 | Yes | F | No Acknowledgment | Not clearly reported | Not stated | No | No Acknowledgment | 2SLS | Regression coefficient | Adjusted | No | Not Stated |
| Painter et al., 2016 | Yes | OR | No Acknowledgment | Not clearly reported | Not stated | No | No Acknowledgment | IVW | OR | Adjusted | No | Not Stated |
| Palmar et al., 2011 | Yes | F, R^2^ | Clearly Stated & Discussed | Not clearly reported | Not stated | No | No Acknowledgment | 2SLS | Regression coefficient, OR | Adjusted | No | Stated |
| Partida et al., 2015 | Yes | F | Clearly Stated & Discussed | Not clearly reported | Not stated | No | No Acknowledgment | 2SLS | Regression coefficient | Adjusted | No | Stated |
| Policicchio et al., 2017 | Yes |  | Clearly Stated & Discussed | Not clearly reported | Not stated | No | No Acknowledgment | IVW | OR | Unadjusted | Yes | Not Stated |
| Postmus et al., 2015 | No |  | No Acknowledgment | Not clearly reported | Not stated | No | No Acknowledgment |  | Regression coefficient | Unadjusted | No | Not Stated |
| Prins et al., 2016 | Yes | F, R^2^ | No Acknowledgment | Not clearly reported | Not stated | No | No Acknowledgment | IVW | OR | Unadjusted | No | Not Stated |
| Rasheed et al., 2014 | Yes | F, R^2^ | Clearly Stated & Discussed | Not clearly reported | Not stated | No | No Acknowledgment | 2SLS | Regression coefficient | Adjusted | No | Stated |
| Reed et al., 2017 | Yes |  | Clearly Stated & Discussed | Not clearly reported | Not stated | No | No Acknowledgment | IVW | Regression coefficient | Adjusted | Yes | Not Stated |
| Rhead et al.,2016 | Yes |  | Clearly Stated & Discussed | Not clearly reported | Not stated | No | Stated but not Discussed 4h |  | OR | Adjusted | No | Not Stated |
| Richmond et al., 2017 | Yes | F, R^2^ | Clearly Stated & Discussed | Not clearly reported | ATE | No | No Acknowledgment | IVW | Regression coefficient | Adjusted | Yes | Not Stated |
| Rodriguez-Broadbent et al., 2017 | Yes |  | Lacked Clear Discussion | Not clearly reported | Not stated | No | No Acknowledgment | Wald Estimator | OR | Unadjusted | No | Not Stated |
| Schmitz et al., 2015 | Yes | F, R^2^ | Clearly Stated & Discussed | Not clearly reported | LATE | No | Stated but not Discussed 4h | 2SLS | Regression coefficient | Unadjusted | Yes | Stated |
| Schnurr et al., 2017 | No |  | Lacked Clear Discussion | Not clearly reported | Not stated | No | No Acknowledgment | 2SLS | Regression coefficient | Adjusted | No | Not Stated |
| Shapland et al., 2018 | Yes | F | Lacked Clear Discussion | Not clearly reported | Not stated | Yes | No Acknowledgment | LIML | Regression coefficient | Unadjusted | No | Not Stated |
| Shi et al., 2018 | Yes |  | No Acknowledgment | Not clearly reported | Not stated | No | No Acknowledgment | IVW | OR | Unadjusted | Yes | Stated |
| Shungin et al., 2015 | No |  | No Acknowledgment | Not clearly reported | Not stated | No | No Acknowledgment | IVW | RR | Adjusted | No | Not Stated |
| Skaaby et al., 2017 | Yes | F | Lacked Clear Discussion | Not clearly reported | Not stated | Yes | No Acknowledgment |  | OR | Adjusted | No | Not Stated |
| Smith et al., 2014 | Yes |  | Lacked Clear Discussion | Not clearly reported | Not stated | No | No Acknowledgment |  | OR | Unadjusted | Yes | Not Stated |
| Taylor et al., 2016 | No |  | No Acknowledgment | Not clearly reported | Not stated | No | No Acknowledgment |  | Regression coefficient | Unadjusted | Yes | Not Stated |
| Thompson et al., 2016 | No |  | No Acknowledgment | Not clearly reported | Not stated | No | No Acknowledgment | IVW | Regression coefficient | Unadjusted | No | Not Stated |
| Thrift et al., 2014 | No |  | No Acknowledgment | Not clearly reported | Not stated | No | No Acknowledgment | 2SLS | OR | Adjusted | No | Not Stated |
| Thrift et al., 2014 | Yes | F | Clearly Stated & Discussed | Not clearly reported | Not stated | Yes | No Acknowledgment | 2SRI | OR | Unadjusted | No | Not Stated |
| Thrift et al., 2015 | Yes |  | No Acknowledgment | Not clearly reported | Not stated | No | No Acknowledgment | Wald Estimator | OR | Adjusted | No | Not Stated |
| Tikkanen et al., 2016 | Yes |  | No Acknowledgment | Not clearly reported | Not stated | No | No Acknowledgment | 2SLS | OR | Adjusted | Yes | Not Stated |
| Trajanoska et al., 2018 | Yes |  | Lacked Clear Discussion | Reported 1 type | Not stated | No | No Acknowledgment | IVW | OR | Unadjusted | No | Not Stated |
| Treur et al., 2018 | No |  | No Acknowledgment | Not clearly reported | Not stated | No | No Acknowledgment | IVW | OR | Unadjusted | Yes | Not Stated |
| Viinikainen et al., 2018 | Yes |  | Clearly Stated & Discussed | Reported 2 or more types | LATE | No | Stated and Discussed 4m | 2SLS | Regression coefficient | Adjusted | No | Not Stated |
| Walter et al., 2015 | Yes | F | Clearly Stated & Discussed | Reported 1 type | Not stated | No | No Acknowledgment | IVW | Regression Coefficient | Adjusted | No | Stated |
| Walter et al., 2015 | Yes | F | Lacked Clear Discussion | Not clearly reported | Not stated | No | No Acknowledgment |  | Regression coefficient | Adjusted | No | Stated |
| Wang et al., 2018 | Yes |  | Clearly Stated & Discussed | Not clearly reported | Not stated | No | No Acknowledgment | IVW | OR | Adjusted | Yes | Not Stated |
| Wang et al., 2018 | Yes |  | Clearly Stated & Discussed | Reported 1 type | Not stated | Yes | No Acknowledgment | Wald Estimator | OR | Adjusted | Yes | Stated |
| Wang et al., 2019 | Yes | F, R^2^ | Clearly Stated & Discussed | Not clearly reported | Not stated | No | No Acknowledgment | Wald Estimator | OR | Adjusted | Yes | Not Stated |
| Wang et al., 2018 | Yes |  | Clearly Stated & Discussed | Reported 1 type | Not stated | Yes | No Acknowledgment |  | Regression coefficient | Adjusted | Yes | Not Stated |
| Wesolowka et al., 2017 | Yes | F | Lacked Clear Discussion | Not clearly reported | Not stated | No | No Acknowledgment |  | Regression coefficient | Adjusted | No | Not Stated |
| Wood et al., 2019 | No |  | No Acknowledgment | Not clearly reported | Not stated | Yes | No Acknowledgment | IVW | OR | Unadjusted | Yes | Not Stated |
| Xiong et al., 2015 | Yes | F, R^2^ | Clearly Stated & Discussed | Not clearly reported | Not stated | No | No Acknowledgment | 2SLS | Regression coefficient | Adjusted | No | Stated |
| Xu et al., 2016 | Yes |  | Clearly Stated & Discussed | Not clearly reported | Not stated | No | No Acknowledgment | Wald Estimator | OR | Adjusted | Yes | Not Stated |
| Xu et al., 2016 | Yes |  | Lacked Clear Discussion | Not clearly reported | Not stated | Yes | No Acknowledgment |  | OR | Adjusted | Yes | Not Stated |
| Xuan et al., 2018 | Yes |  | Clearly Stated & Discussed | Not clearly reported | Not stated | No | No Acknowledgment | 2SLS | OR | Adjusted | Yes | Stated |
| Yaghootkar et al., 2013 | Yes | F | No Acknowledgment | Not clearly reported | Not stated | No | No Acknowledgment | 2SLS | OR | Adjusted | Yes | Not Stated |
| Yan et al., 2016 | Yes |  | No Acknowledgment | Not clearly reported | Not stated | No | No Acknowledgment | 2SLS | OR | Adjusted | No | Stated |
| Zeng et al., 2019 | Yes | F | No Acknowledgment | Not clearly reported | Not stated | No | No Acknowledgment |  | OR | Adjusted | Yes | Not Stated |
| Zhan et al., 2017 | No |  | No Acknowledgment | Not clearly reported | Not stated | No | No Acknowledgment | 2SLS | OR | Unadjusted | Yes | Not Stated |
| Zwakenberg et al., 2019 | No |  | Lacked Clear Discussion | Not clearly reported | Not stated | No | No Acknowledgment |  | RR | Adjusted | No | Stated |
